# Pharmacodynamic Model of the Hemodynamic Effects of Propofol and Remifentanil and Their Interaction with Noxious Stimulation †

**DOI:** 10.3390/pharmaceutics16121615

**Published:** 2024-12-19

**Authors:** Maite Garraza-Obaldia, Sebastian Jaramillo, Zinnia P. Parra-Guillen, José F. Valencia, Pedro L. Gambús, Iñaki F. Trocóniz

**Affiliations:** 1 Department of Pharmaceutical Sciences, School of Pharmacy and Nutrition, University of Navarra, 31008 Pamplona, Spain; zparra@unav.es (Z.P.P.-G.); itroconiz@unav.es (I.F.T.); 2 Navarra Institute for Health Research (IdiSNA), 31008 Pamplona, Spain; 3 Anesthesiology Department, Hospital CLINIC de Barcelona, 08036 Barcelona, Spain; 4 Biomedical Engineering Program, Universidad de San Buenaventura, Cali 111321, Colombia; jfvalenc@usbcali.edu.co; 5 Institut d‘Investigacions Biomèdiques Agusti Pi i Sunyer (IDIBAPS), 08036 Barcelona, Spain; 6 Institute of Data Science and Artificial Intelligence (DATAI), University of Navarra, 31008 Pamplona, Spain

**Keywords:** PKPD modelling, population approach, anesthesia, mean arterial pressure, heart rate, hysteroscopy, clinical routine

## Abstract

**Background**: Despite the known impact of propofol and remifentanil on hemodynamics and patient outcomes, there is a lack of comprehensive quantitative analysis, particularly in surgical settings, considering the influence of noxious stimuli. The aim of this study was to develop a quantitative semi-mechanistic population model that characterized the time course changes in mean arterial pressure (MAP) and heart rate (HR) due to the effects of propofol, remifentanil, and different types of noxious stimulation related to the clinical routine. **Methods**: Data from a prospective study were used; the study analyzed the effects of propofol and remifentanil general anesthesia on female patients in physical status of I-II according to the American Society of Anesthesiologists (ASA I-II) undergoing gynecology surgery. Patients were consecutively assigned to different administration schemes of propofol and remifentanil targeted at different effect-site concentrations. Esophageal instrumentation, laryngeal mask airway insertion, hysteroscopy, and tetanus stimuli were applied. Data from patients with chronic hypertension were discarded. **Results**: MAP and HR observations from 77 patients were analyzed. The hemodynamic effects were described using turn-over models incorporating feedback mechanisms. Analyses revealed that propofol and remifentanil elicited effects on the turn-over of MAP and HR, respectively, with estimates of plasma drug concentrations causing an inhibition-half of the maximum effect (*C*50) of 8.79 µg∙mL^−1^ and 4.57 ng∙mL^−1^. Hysteroscopy exerted an increase in MAP (but not in HR), which was well-characterized by the model, with a predicted typical increase of 28 mmHg and a dissipation half-life of 33 min. The impact of other noxious stimuli on MAP or HR could not be identified. Model simulations indicated that propofol and remifentanil, titrated to inhibit the motor response to noxious stimuli, regardless of dose combinations, cause a significant risk of hypotension, especially following induction and at the end of surgery (when surgical intervention is completed, before the awakening phase). **Conclusions**: The developed semi-mechanistic and fully identifiable model provides quantitative information on how propofol, remifentanil, and surgical stimulus (hysteroscopy) interact to produce the hemodynamic changes (of MAP and HR) commonly observed in clinical practice.

## 1. Introduction

Hemodynamic changes during sedation and general anesthesia are common and potentially harmful [1,2]. One of the driving factors is anesthetic drugs, such as propofol and remifentanil, which are administered to protect the patient from the changes that represent surgical intervention and counteract the effects of noxious stimulation.

When drug administration is below requirements, the patient might develop hemodynamic stress reactions to noxious stimulation, including tachycardia and arterial hypertension, which may evolve into specific adverse events (such as myocardial ischemia, arrhythmia, cerebrovascular accident, left ventricular failure, and rupture of pre-existing aneurysms or atherosclerotic vessels, among others) [1]. On the contrary, a too intense reaction to the anesthetic drugs might cause a fall in arterial blood pressure that can affect organ perfusion and eventually evolve to the malfunction of different organs and systems [2]. Besides the pharmacologic effects of anesthetic drugs, the occurrence of hemodynamic changes depends also on the type and magnitude of noxious stimulation.

The effects of propofol and remifentanil on hemodynamic variables have been quantitatively analyzed using different approaches [3,4,5,6]. However, these effects were described in the absence of noxious stimulation. It remains unclear how the effects of these drugs interact with noxious stimulation to produce the hemodynamic changes commonly observed in clinical practice. In light of the findings associating hemodynamic changes with unfavourable outcomes, it is crucial to understand the relationship between anesthetics, noxious stimulation, and hemodynamic variables in quantitative terms in order to design dosing strategies to reduce the risk of significant hemodynamic changes (e.g., mean arterial pressure below 60 mmHg or variations greater than 20% from baseline levels).

The aim of this study was to develop a fully identifiable population model that characterized the changes in mean arterial pressure (MAP) and heart rate (HR) due to the effects of propofol, remifentanil, and different types of noxious stimulation during clinical practice. The developed model was further used to explore the effects of propofol and remifentanil combinations in simulated clinical scenarios.

## 2. Materials and Methods

### 2.1. Study Design

After obtaining written informed consent, data were prospectively collected from 80 consecutive patients undergoing outpatient gynecologic surgeries. The data collected became part of the physiologic signal analysis under the general anesthesia database, authorized by the Institutional Review Board of Hospital CLINIC de Barcelona, Spain (reference 2013/8356; date of approval 2 July 2013). Inclusion criteria were ASA I-II, whereas exclusion criteria were BMI > 35 kg∙m^−2^, cardiovascular diseases other than chronic hypertension, respiratory diseases, neurological diseases, use of psychotropic drugs, and drug abuse.

No premedication was administered. Once demographic data were recorded, an intravenous catheter was placed in the cubital fossa and oxygen was administered through a nasal cannula. HR was measured through three-lead continuous electrocardiography, and MAP was measured noninvasively through oscillometry every 1 min, both by a patient monitor (Intellivue MX500; Philips, Amsterdam, The Netherlands). Other monitoring measures included pulse oximetry, capnography through nasal cannula, and bispectral index (BIS Vista; Covidien, Boulder, CO, USA).

Upon arrival to the operating room, patients were consecutively assigned to a propofol and remifentanil scheme, which consisted of a 25 min infusion targeted at effect-site concentrations in a step-up fashion, followed by a 15 min step-down infusion. The study protocol comprised 4 groups. In each group, one of the two drugs was either not administered or targeted at a certain concentration while the other was varied. Patients in Group A received only propofol, patients in Group B received only remifentanil, patients in Group C received an infusion targeted at stable propofol and varying remifentanil concentrations, and patients in Group D received an infusion targeted at stable remifentanil and varying propofol concentrations. For each group, 8 to 14 different schemes of propofol and remifentanil were considered, each with a different rate of change in target concentrations. The variety of changes in the concentration of propofol and remifentanil was intended to perturb the biological system and thereby gather the observations required to develop a semi-mechanistic model. The schemes are detailed in Table S1.

Propofol and remifentanil were administered via target-controlled infusion (Base Primea Orchestra; Fresenius Kabi, Brézins, France) based on the pharmacokinetic–pharmacodynamic models of Schnider (with a *k_e_*_0_ of 0.456 min^−1^) [7] and Minto [8], respectively. In each step, the target concentrations were kept stable for at least 5 min. In the case of respiratory depression, the patient was ventilated through a facemask. If patients remained conscious after the first 25 min, the propofol target concentration was increased by 1 μg∙mL^−1^ every 5 min. After loss of consciousness, an attempt was made to place a laryngeal mask (LMA). If the patient presented movement or resistance, the remifentanil target concentration was increased by 0.5 ng∙mL^−1^ and a further attempt was made every 5 min. After placement of the LMA, the target concentrations were either kept or reduced in stepwise fashion for another 15 min, according to the predefined schemes. Propofol target concentrations could not be reduced if the BIS index was equal to or greater than 60. After 15 min, target concentrations were kept stable until the start of surgery. After the start of surgery, the attending anesthesiologist could adjust target concentrations as deemed necessary.

This protocol was replicated for Groups A, C, and D, incorporating noxious stimulation. Once patients lost consciousness, a noxious stimulus was applied at the end of each step, and target concentrations were kept stable during an additional 2 min to observe any response to the stimulus. First, an esophageal stimulus (esophageal instrumentation) was applied, which consisted of attempting to introduce a 7.0 mm endotracheal tube (which has an outer diameter of approximately 10 mm) 25 cm through the oropharynx and esophagus. If the patient presented response in the form of movement, gag reflex, or increase in >20% of HR or MAP within 2 min, the stimulus was applied again at the end of the next step. In the case of no response to the esophageal stimulus, tetanic stimuli were applied in the following steps. Tetanic stimuli were performed using a pupilometer (Algiscan; Idmed, Marseille, France) with leads connected to the right forearm, and applying a current of 60 mA for 5 s. If the patient presented a response in the form of movement or hemodynamic changes in the descending phase steps, no further stimulus was applied in the subsequent steps.

### 2.2. Data Handling

For the present study, data from patients with chronic hypertension were discarded, since they represented less than 1% of the available data. Data from patients undergoing hysteroscopy or other types of interventions were analyzed up to emergence. Data were also censored after the administration of atropine (IV), ephedrine (IV), or any other vasoactive drug (as norepinephrine or dopamine).

All data were collected and synchronized using VitalRecorder software (the program is freely downloadable from the website, https://vitaldb.net; last accessed on 13 October 2022) [9]. Data sampling was reduced to 1 data point every 10 s for infusion data, while data sampling for HR and MAP was kept by 1 data point every 1 min. The onset of MAP and HR measurements was collected prior to the administration of propofol and remifentanil anesthesia for all patients.

Patients were distributed into training and test groups for model development and validation, respectively, based on the surgical procedure they underwent. The training group involves patients undergoing hysteroscopy and the test group involves patients undergoing other types of surgical procedures.

### 2.3. Statistical Analyses

The time course of the MAP and HR observations were analyzed simultaneously through the nonlinear mixed effects modelling approach, using the first-order conditional estimation method with interaction available in NONMEM (NONMEM version 7.4; Icon Development Solutions, Hanover, MD, USA).

Selection between model candidates was made based on the following criteria: (i) comparison of the minimum objective function value, approximately equal to −2∙log (likelihood) (−2 LL). For two nested models differing in one parameter, a reduction in −2 LL of 3.84 or 6.61 points corresponds to significance levels of 5 and 1%, respectively. (ii) Precision of the parameter estimates as quantified by the relative standard errors (RSE), calculated as the ratio multiplied by 100 between the standard error and the point estimate of the parameter. (iii) Visual inspection of the goodness of fit plots.

Model building followed three steps. First, a base population model providing an adequate description of the data were developed. At this step, potential covariance between random effects was explored for significance. Then, the impact of covariates on model parameters was investigated. Finally, the selected model was evaluated through simulation-based diagnostics.

### 2.4. Model Development

The base model was developed as a simplified version of the models proposed by Snelder et al. [10] and Fu et al. [11], and taking into consideration the mechanisms described in the models of Jeleazcov et al. [3] and Su et al. [5,6]

The structure of the model is based on turn-over mechanisms [12], where the rate of change in the response variables (*dMAP*/*dt* and *dHR*/*dt*) are governed by input and degradation rates, respectively, the physiological feedbacks between the two variables, and the external perturbations corresponding to drug concentrations and noxious stimulation, as indicated in Equations (1) and (2).

(1) $$\frac{dMAP}{dt}=k_{in,MAP}\times \left(1-f\left(Propo\right)\right)\times \left(1-g_{MAP}\left(Remi\right)\right)\times p\left(HR\right)\times h_{MAP}\left(Stim\right)-k_{out,MAP}\times MAP$$

(2) $$\frac{dHR}{dt}=k_{in,HR}\times \left(1-g_{HR}\left(Remi\right)\right)\times q\left(MAP\right)\times h_{HR}\left(Stim\right)-k_{out,HR}\times HR\;$$

where *k_in_*_,*i*_ and *k_out_*_,*i*_ are the zero-order and first-order input and degradation rate constants, respectively, and the subscript *i* denotes the type of response (HR or MAP). *k_in_*_,*i*_ are parameters derived from Equations (1) and (2) under steady-state conditions. *f* and *g_i_* are the functions that represent the effects of propofol and remifentanil on each response, respectively. It was initially considered that propofol could only exert an effect on MAP, whereas it was proposed that remifentanil could affect both MAP and HR, and that this effect could differ between the two variables. *p* and *q* are the functions that represent the feedback mechanisms of HR on MAP and of MAP on HR, respectively. Finally, *h_i_* is the function that represents the effect of noxious stimulation for *i* response. The initial conditions of the model are represented by the model parameters *MAP*_0_ and *HR*_0_, defined at the MAP and HR values previous to drug administration.

The functions *f*(*Propo*) and *g_i_*(*Remi*) were expressed as follows:

(3) $$f\left(Propo\right)=\frac{{Emax}_{Propo}\times {C_{Propo}}^{\gamma_{Propo}}}{{C_{Propo}}^{\gamma_{Propo}}+{{C50}_{Propo}}^{\gamma_{Propo}}}$$

(4) $$g_{i}\left(Remi\right)=\frac{{Emax}_{Remi,i}\times {C_{Remi}}^{\gamma_{Remi,i}}}{{C_{Remi}}^{\gamma_{Remi,i}}+{{C50}_{Remi,i}}^{\gamma_{Remi,i}}}$$

where *Emax_Propo_* and *Emax_Remi_*_,*i*_ are the maximum fractional inhibition of propofol on MAP and remifentanil on *i* response, respectively. *C_Propo_* and *C_Remi_* are the predicted plasma concentration of propofol and remifentanil, respectively. *C*50*_Propo_* and *C*50*_Remi_*_,*i*_ are the plasma concentration of propofol and remifentanil that cause half of the Emax effect, and the parameters *γ_Propo_* and *γ_Remi_*_,*i*_ control the steepness of the concentration effect curves [13]. *Emax_Propo_* and *Emax_Remi_*_,*i*_ parameters were constrained to values between 0 and 1. According to Equations (1) and (2), both drugs have inhibitory effects on hemodynamic variables. Further analyses were performed to investigate whether effect-site propofol and remifentanil concentrations were better drivers of the hemodynamic response than the levels in plasma, estimating the corresponding *k_e_*_0_ parameter. Also, an interaction between propofol and remifentanil in Equation (1) was tested.

The known tight regulation within the cardiovascular system is represented in the model by the functions *p*(*HR*) and *q*(*MAP*), which were expressed as follows:

(5) $$p\left(HR\right)={\left(\frac{HR}{{HR}_{0}}\right)}^{{FB}_{HR}}$$

(6) $$q\left(MAP\right)={\left(\frac{MAP}{{MAP}_{0}}\right)}^{-{FB}_{MAP}}$$

where *FB_HR_* and *FB_MAP_* are the parameters that modulate the relationship between HR and MAP and their corresponding baseline value.

The function *h_i_*(*Stim*) was expressed as follows:

(7) $$h_{i}\left(Stim\right)=\left(1+\theta_{Stim,i}\times e^{-k_{Stim,i}\times (t-t_{Stim})}\right)$$

where *t_Stim_* is the time at which the noxious stimulus starts, *k_Stim_*_,*i*_ represents the first-order rate of the disappearance of the effect, and *θ_Stim_*_,*i*_ is the parameter accounting for the impact of the stimulus on *k_in_*_,*i*_. At *t* ≤ *t_sg_*, *h_i_*(*Stim*) = 1.

In the absence of external perturbation due to propofol, remifentanil, or stimuli, and ignoring circadian variations (i.e., steady-state conditions, where *dMAP*/*dt* and *dHR*/*dt* are equal zero), the functions *f*(*Propo*), *g_i_*(*Remi*), and *h_i_*(*Stim*) take the value of 1; therefore, *k_in_*_,*MAP*_ = *k_out_*_,*MAP*_ × *MAP*_0_ and *k_in_*_,*HR*_ = *k_out_*_,*HR*_ × *HR*_0_.

Interindividual variability (IIV) was modelled assuming log-normal distributions of model parameters, with the exception of *Emax_Propo_* and *Emax_Remi_*_,*i*_, in which logistic models were used to constrain values between 0 and 1. MAP and HR residual errors were modelled with additive models.

The selection of the covariates was performed using the stepwise covariate model building method, with levels of significance of 0.05 and 0.01 for the forward selection and backward deletion of covariates, respectively [14]. The covariates explored for statistical significance on model parameters were age, weight, body mass index, surgery duration (continuous), changes in patient position, and the type of noxious stimulus (categorical). Surgical stimulus (hysteroscopy) was considered the reference stimulus in the modelling of the noxious stimuli.

### 2.5. Model Evaluation and Validation

Model evaluation was performed through simulation-based model diagnostics, including the goodness of fit plots and prediction-corrected visual predictive checks (pcVPC). pcVPC were performed simulating 500 datasets with the same characteristics as the original study, and correct the observations and simulated values by the median of the population prediction for each time bin [15].

Parameter precision was further investigated analyzing 500 bootstrap datasets and computing the 95% confidence intervals of the parameter distributions.

The test dataset was used for validation, generating a pcVPC to evaluate model performance.

### 2.6. Simulations

**Figure 1:**
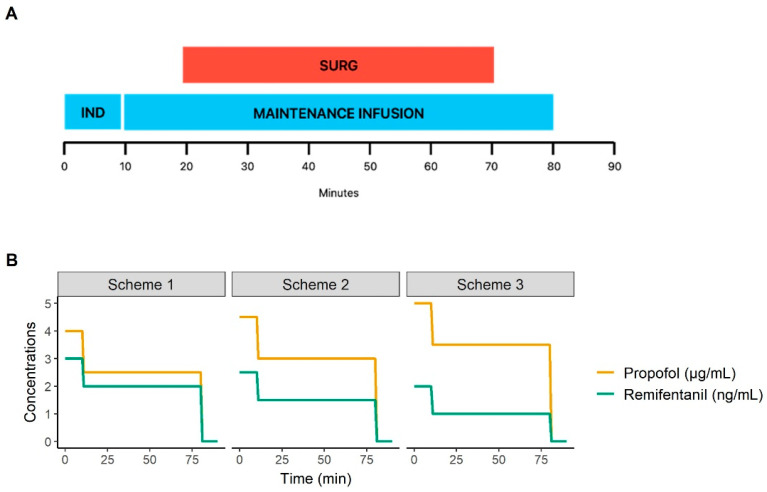
A schematic representation of the simulated scenario and administration schemes. (**A**) The scenario simulated a 90 min surgical procedure, involving LMA insertion at 5 min, and surgical stimulation (hysteroscopy) from 20 to 70 min. (**B**) Propofol and remifentanil infusions were simulated using a target-controlled infusion based on the models described by Schnider and Minto, respectively, and set at varying target effect-site concentrations. The induction targets were maintained until 10 min, then the maintenance targets were maintained until 80 min, and finally the targets were set to zero. Three schemes were simulated with the following effect-site concentration targets: (i) propofol at 4.0 μg∙mL^−1^ and remifentanil at 3.0 ng∙mL^−1^ for induction, and propofol at 2.5 μg∙mL^−1^ and remifentanil at 2.0 ng∙mL^−1^ for maintenance; (ii) propofol at 4.5 μg∙mL^−1^ and remifentanil at 2.5 ng∙mL^−1^ for induction, and propofol at 3.0 μg∙mL^−1^ and remifentanil at 1.5 ng∙mL^−1^ for maintenance; and (iii) propofol at 5.0 μg∙mL^−1^ and remifentanil at 2.0 ng∙mL^−1^ for induction, and propofol at 3.5 μg∙mL^−1^ and remifentanil at 1.0 ng∙mL^−1^ for maintenance.

Stochastic simulations were performed to explore the impact of three propofol and remifentanil schemes on MAP in a general anesthesia scenario, involving LMA insertion and a hysteroscopy procedure. The details of the simulated scenario and the three schemes of administration are presented in Figure 1. The three administration schemes were designed to achieve probabilities of non-response to LMA insertion and tetanic stimulation close to 70%, based on the models described by Zaballos et al. and Kern et al., respectively [16,17].

For each administration scheme, one thousand MAP profiles were simulated, and the probabilities of hypotension were calculated. Hypotension was defined as (i) a MAP value below 60 mmHg and (ii) a decrease of at least 20% in MAP from the baseline value estimated by the model.

### 2.7. Software and Tools

Data filtering, formatting, and graphical representation were performed with R version 4.0.2 using RStudio v2021.09.2 (Posit, Boston, MA, USA). Simulation-based diagnostics, covariate evaluation, and bootstrap simulations were performed with the support of Perl-Speaks-NONMEM [18,19].

## 3. Results

Of the 80 patients in the database, 3 were excluded due to chronic arterial hypertension. The characteristics of the 77 patients available for analysis are shown in Table 1. The training group comprised 56 patients for whom response measures during hysteroscopy surgery were available. The test group consisted of 21 patients; their pre-surgery data were used to validate the general anesthesia induction and the period prior to surgical stimulation, while data from no hysteroscopy surgeries (available in eight of the twenty-one patients) were also used for model validation during surgery. The 21 patients (27%) had at least 1 MAP measurement < 65 mmHg; 4 in Group A, 8 in Group B, 6 in Group C, and 3 in Group D. No record was censored due to the administration of vasoactive drugs. Raw data of MAP, HR, and the predicted propofol and remifentanil plasma concentrations are displayed in Figure S1 for randomly selected individuals. The characteristics of the patients in each group are summarized in Table 1. Regarding changes in patient position during the procedure, three options were noted: supine, lithotomy, and Trendelenburg. There were 6% of patients always in the supine position, 76% had some change in position from supine, and 18% had all three positions.

### 3.1. Model Development

During model development, results indicated that the current data did not support the inclusion of the drug effect-site compartment model, nor a direct effect of remifentanil on MAP (*p* > 0.05), the latter supported by the lack of visually perceptible changes in MAP in patients during the infusion of remifentanil alone. Estimates of *Emax_Propo_* and *Emax_Remi_*_,*HR*_ were significantly different from both 0 and 1 (*p* < 0.01). The estimate of the *γ_Propo_* parameter in Equation (3) was not different than 1 (*p* > 0.05); therefore, it was removed from the model. No evidence of significant pharmacodynamic interaction between propofol and remifentanil on MAP was detected during model development (*p* > 0.05).

The effect of HR on MAP significantly improved model fit (*p* < 0.01), with the related parameter, *FB_HR_*, estimated to be different from 0 and 1 (*p* < 0.01). On the contrary, the *FB_MAP_* parameter, which modulates the impact of MAP on HR, was estimated to be close to zero with very low precision and was therefore not included in the final model.

Surgical stimulus (hysteroscopy) was found to impact MAP but not HR (*p* > 0.05). The time course of the surgical stimulus was described as a monoexponential decay, with a maximum value at the onset of the stimulus. Other models based on the Bateman function were also tested without obtaining a significant better fit (*p* > 0.05). The estimate obtained for the variability associated with the dissipation rate constant of the surgical stimulus was unrealistically high and not related with the duration of the surgical procedure but driven mainly by a small number of patients (n = 13). Thus, it was decided to use a fixed estimate of the variance equivalent to a 100% coefficient of variation, without compromising individual predictions and with minor variation in typical population estimates.

No significant impact of the other noxious stimuli (LMA insertion, esophageal instrumentation, and tetanic stimulation) on MAP or HR could be identified, as was evaluated by both *k_Stim_* and *θ_Stim_* parameters in Equation (7). Nor did position changes during the procedure show a significant impact on the evaluated responses.

None of the covariates analyzed showed a significant relationship with any of the parameters of the model (*p* > 0.05).

**Figure 2:**
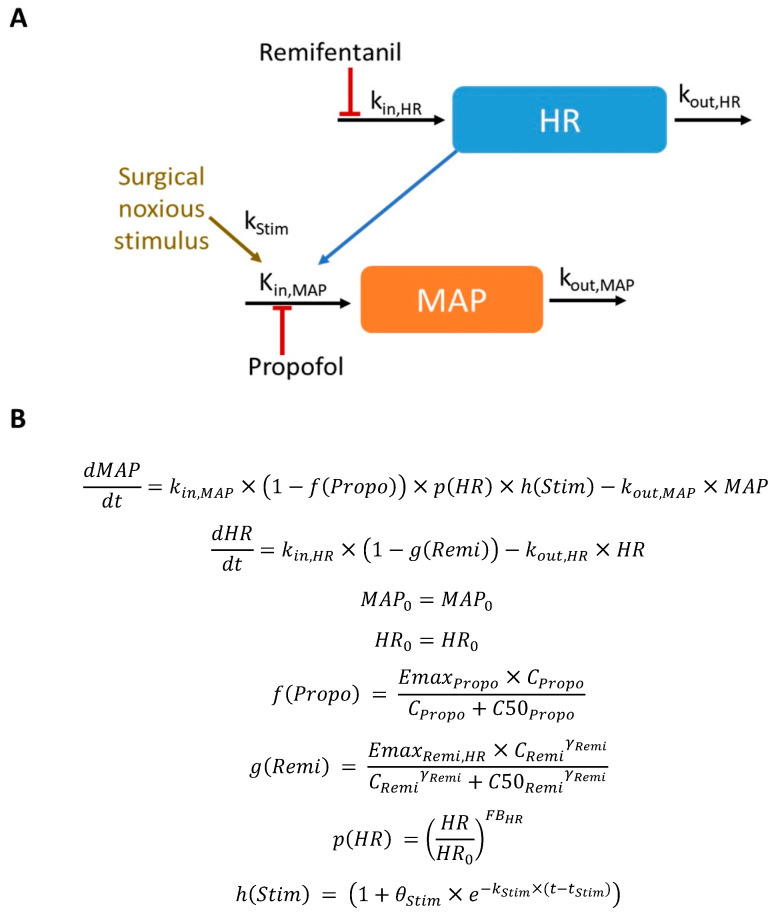
A representation of the final model. Schematic (**A**) and mathematical (**B**) representations of the final model. Propofol and remifentanil exert their inhibitory effects *f*(*Propo*) and *g*(*Remi*) on the zero-order input rate constant of MAP or HR (*k_in_*_,*MAP*_ and *k_in_*_,*HR*_, respectively). *C_Propo_* and *C_Remi_* are the predicted propofol and remifentanil plasma concentrations, respectively. *Emax* is the maximum fractional inhibition that the drug can exert, while *C*50 is the predicted plasma concentrations of the drug that causes an inhibition-half of *Emax*. The parameter *γ_Remi_* controls the steepness of the inhibition induced by remifentanil. *k_out_* is the first-order rate constant of response degradation. Feedback between the responses is represented by the term *p*(*HR*), driving relative changes in HR with respect to its baseline (*HR*_0_) nonlinearly (*FB_HR_*) on MAP. The term *h*(*Stim*) accounts for the temporal impact exerted by the noxious stimulus on MAP, where *t_Stim_* is the time at which surgery procedure starts, *k_Stim_* represents the first-order rate constant of disappearance, and *θ_Stim_* accounts for the impact of surgery on *k_in_*_,*MAP*_.

**Figure 3:**
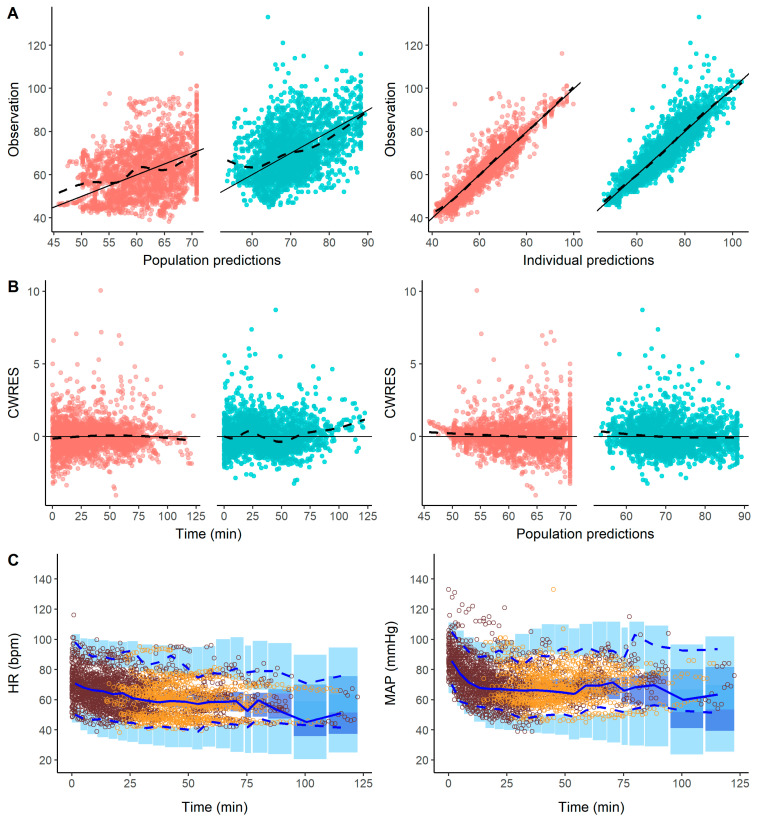
Model evaluation. Goodness of fit plots: (**A**) HR and MAP observations vs. population and individual predictions; (**B**) conditional weighted residuals (CWRES) vs. time and population predictions. For panels (**A**,**B**), red and blue solid circles represent HR and MAP, respectively, while solid lines represent the perfect fit and the dashed lines show the loess smoothing curve through the data. Units of measurement for observations and predictions (population and individual): MAP in mmHg, HR in bpm. (**C**) Prediction-corrected visual predictive checks for MAP and HR. The median (solid line) and 2.5th and 97.5th percentiles (dashed lines) of the raw data are plotted along with the 95% prediction intervals (shaded area) for those same percentiles obtained from 500 simulated datasets. The light blue areas represent the 95% prediction intervals for the 2.5th and 97.5th percentiles, while the dark blue area represents the 95% prediction interval for the 50th percentile. Observations before and after the surgical intervention are coloured in brown, while observations during the stimulation are coloured in yellow.

Schematic and mathematical forms of the final model are represented in Figure 2. Table 2 lists the estimates of the final model. Most of parameters were estimated with good precision (RSE < 40%). The low values of shrinkage (< 15%) reported in Table 2 indicate that estimates of the individual model parameters are reliable and the observations vs. individual predictions for the goodness of fit plot are informative and can be used to judge model performance [20]. Goodness of fit plots presented in Figure 3 showed no significant deviations, indicating proper model performance. No trends were detected as a function of time, supporting invariant parameters. Individual fits are presented in Figure S2 for a random selection of individuals.

The typical population estimates of *k_out_*_,*i*_ were not significantly different between MAP and HR (*p* > 0.05). However, the IIV associated with *k_out_*_,*MAP*_ and *k_out_*_,*HR*_ was 143% and 130%, respectively, with both being significantly different (*p* < 0.05). IIV for *MAP*_0_ and *HR*_0_ was low (8.6% and 14.4%, respectively). The derived values for *k_in_*_,*MAP*_ and *k_in_*_,*HR*_ were 10.8 mmHg∙min^−1^ and 8.7 bpm∙min^−1^, respectively. Significant correlation was found between random effects associated with *C*50*_Propo_* and *k_out_*_,*MAP*_ (*p* < 0.01). Covariance between *MAP*_0_ and *HR*_0_ could not be detected.

### 3.2. Model Evaluation

The results of the pcVPC shown in Figure 3 indicate that the model characterized the typical profiles and the dispersion of MAP and HR data well, as well as during the induction and maintenance of general anesthesia.

For none of the model parameters (not even for those with higher RSE), the lower limit of the corresponding 95% confidence intervals obtained from the bootstrap analysis include the value of zero (Table 2).

### 3.3. Model Validation

Figure S3 shows the results from validation. The model developed with the training group data successfully characterized the observations of MAP and HR in the test group.

### 3.4. Model Simulations

**Figure 4:**
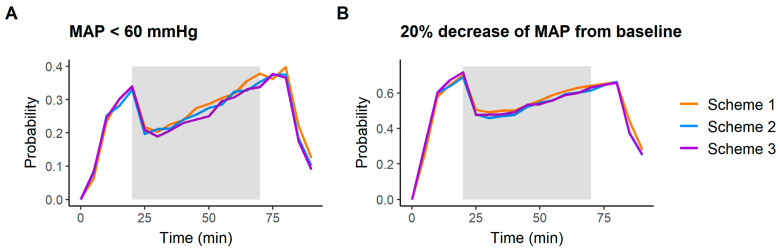
The simulation of the hypotension risk. One thousand simulations were performed for each administration protocol described in Figure 1 to calculate the probabilities of achieving hypotension defined as (**A**) MAP below 60 mmHg and (**B**) a 20% decrease in MAP from baseline. The grey area highlights the period of surgery.

Figure 4 shows the impact of the three different administration schemes on the time course of MAP. Results show negligible differences in MAP across the administration schemes. Before surgery, the maximum probability of hypotension (defined by the criteria of MAP < 60 mmHg and a ≥ 20% decrease from estimated baseline) was 0.35 and 0.70, respectively, occurring just before the start of surgery. During and after surgery, the maximum probability of hypotension was 0.38 and 0.65, respectively, occurring at the end of surgery (when surgical intervention is completed, before the awakening phase). The continuous increase in these probabilities during surgery was the result of the disappearance of the impact of surgical noxious stimuli, as formulated in Equation (7). In this equation, it is assumed that the impact of the stimulus is instantaneous, with higher impact at administration time (the highest increase in MAP) and then decreasing with a first-order rate *k_Stim_* of 0.021 min^−1^.

## 4. Discussion

In this study, we developed an identifiable semi-mechanistic model that accurately described the time course of MAP and HR during the infusion of propofol and remifentanil, as well as in response to surgical stimulus (hysteroscopy). We used a turn-over model framework to describe the hemodynamic effects, which integrated input and degradation rate constants for HR and MAP, as well as a term reflecting the impact of HR on MAP. Analyses suggested that propofol affected MAP, whereas remifentanil exerted effects on HR, and thereby indirectly affected MAP. Analyses also indicated that remifentanil had no direct action on MAP, and that MAP feedback on HR was completely abolished by the effect of propofol. Surgical stimulus exerted an increase in MAP (but not in HR) with a predicted typical increase of 28 mmHg in MAP and a dissipation half-life of 33 min. As a potential explanation for the high variability found in the effect of the noxious stimulus, we cannot rule out the possibility that surgical procedures influenced the change in the hemodynamic response. The impact of other noxious stimuli (esophageal, LMA insertion, and tetanus) on hemodynamic variables could not be identified.

According to the developed model, propofol and remifentanil, titrated to inhibit motor responses to noxious stimuli, cause a significant risk of hypotension, especially following induction and at the end of surgery. Moreover, simulations indicated that the risk of hypotension remains essentially the same despite the use of different combinations of propofol and remifentanil with the same potential to inhibit the motor response to noxious stimuli. Thus, preventing hypotension during propofol and remifentanil anesthesia may require additional actions other than adjusting anesthetics doses, such as the administration of fluid or vasopressors.

The effects of propofol on MAP were modelled using different approaches. Wu et al. described changes in MAP using a direct response model with one effect-site compartment [4], whereas Jeleazcov et al. used a direct response model with two effect-site compartments [3]. Jeleazcov et al. justified the integration of two effect-site compartments on the basis of two distinct pathways of action of propofol (e.g., effects on peripheral vascular resistance and cardiac output), although they did not discard the possibility that one of the compartments reflected the impact of cardiovascular system reflexes [3]. Su et al. developed a mechanism-based model in which propofol had direct effects on peripheral vascular resistance and stroke volume [5]. In addition, the model included MAP close feedback mechanisms on HR, stroke volume, and peripheral vascular resistance. Su et al. demonstrated that the mechanism-based model had a predictive ability indistinguishable from an empirical model based on indirect responses [5].

Previous studies have demonstrated that propofol induces a dose-dependent reduction in MAP [21]. However, the underlying mechanisms remain controversial. While the decrease in MAP was traditionally attributed to a decrease in cardiac output [22,23], studies in both animals and humans have concluded that cardiac output remains unaffected across various propofol doses [21,24,25]. Although propofol induces a reduction in mean systemic filling pressure [26,27], the concurrent decrease in resistance for venous return appears to counteract this, resulting in stable cardiac output due to a hypothetical improved cardiac function [25]. Consequently, in the absence of hypovolemia, propofol appears to reduce MAP mainly by reducing systemic vascular resistance through an inhibition of sympathetic activity [24,28,29,30,31]. These findings disagree with the model of Su et al., which assumes that propofol has a direct effect on stroke volume [5]. Furthermore, to estimate the effect of propofol, Su et al. used pulse pressure as a surrogate for stroke volume, which may not accurately reflect stroke volume in this context, considering the variations in HR and the alteration of arterial compliance due to propofol. Contrary to the models described by Jeleazcov et al. and Su et al. [3,5], our model assumes that propofol affects MAP through only one pathway, which might be related to its effect on systemic vascular resistance.

As for the intrinsic regulation of the cardiovascular system, our analyses supported the inclusion of HR feedback on MAP but not MAP feedback on HR. The former accounts for the effects of HR on cardiac output, whereas the latter is related to the baroreceptor reflex and other regulatory mechanisms. In contrast to the model of Su et al. [5], our analyses support the previous findings indicating that baroreceptor reflex is significantly inhibited, if not abolished, by propofol [29,30,31,32], which further potentiates the hypotensive effects of this drug.

Changes in HR and MAP associated with remifentanil are normal in clinical practice [33,34]. Studies indicate that, given at therapeutic doses, remifentanil causes a decrease in HR [33,34], probably due to an increase in vagal tone [34,35,36]. However, MAP reduction associated with remifentanil remains poorly defined. Studies in humans and animals indicate that remifentanil induces hypotension mainly as a result of the decrease in HR [34,35], while other studies attribute the hypotension to its vasodilator effect [37]. Although studies have demonstrated the vasodilator effect of remifentanil both in vivo and in vitro [38,39], its impact seems to be limited regionally to the site of injection [38], and its impact on MAP may be significant primarily in critically ill patients [37].

Based on their work on propofol-induced hemodynamic changes, Su et al. expanded their model to include the hemodynamic effects of remifentanil [6]. Similarly to our model, the model proposed by Su et al. assumes that remifentanil exerts a direct effect on HR [6]. However, in contrast to our model, theirs also suggests a direct effect on systemic vascular resistance and stroke volume, as well as an interaction with propofol affecting HR, systemic vascular resistance, and stroke volume.

Our study has several limitations. First, the study protocol was mainly designed with the aim of modelling the probability of motor response to noxious stimulation. With this protocol, noxious stimuli were applied at concentrations of propofol and remifentanil higher than those that might elicit hemodynamic responses, which prevented us from identifying changes in MAP induced by esophageal stimulation, LMA insertion, and tetanus. However, it is also possible that the magnitude of these stimuli may not be significant enough to provoke a hemodynamic response, at least in the context of unconsciousness. Second, the plasma concentrations of propofol and remifentanil used in this study were predicted and not actually measured. Having measured plasma concentrations would have allowed us to exclude potential differences with population predictions and to ensure that the concentration prediction is not affected by other players. Third, we did not measure stroke volume nor systemic vascular resistance, and we did not analyze systolic and diastolic pressures. Although our semi-mechanistic approach did not contemplate the description of changes in these variables, their measurement and analysis would have allowed us to confirm the mechanisms underlying changes in MAP.

Preliminary results of this study were presented as a poster at 31st Annual Meeting of the Population Approach Group in Europe [40].

## 5. Conclusions

In conclusion, we developed a model that accurately described the changes in HR and MAP induced by propofol, remifentanil, and hysteroscopy stimulus. The developed model provides quantitative information on how propofol, remifentanil, and surgical stimulus interact to produce the hemodynamic changes commonly observed in clinical practice. Simulations performed with the model indicate that the administration of propofol and remifentanil, titrated to inhibit motor responses to noxious stimuli, cause a significant risk of hypotension, particularly following induction and at the end of surgery. This risk persists consistently across various combinations of these agents with the same potential to inhibit the motor response to noxious stimuli. Thus, preventing hypotension during propofol and remifentanil anesthesia may require additional actions other than adjusting anesthetics doses, such as the administration of fluid or vasopressors.

## Figures and Tables

**Table 1:** The characteristics of the patients available for the analysis (n = 77).

	Training Group (n = 56)	Test Group (n = 21)	Total (n = 77)
Age (years), mean (SD)	42 (11)	47 (15)	43 (12)
Height (m), mean (SD)	1.61 (7)	1.61(7)	1.61 (7)
Weight (kg), mean (SD)	62 (9)	64 (11)	62 (10)
BMI (kg∙m^−2^), mean (SD)	24 (4)	25 (4)	24 (4)

**Table 2:** The parameter estimates of the final model and their corresponding precision.

Parameters	Estimate (RSE %)	Median (95% CI)	Shrinkage (%)
Drug related			
*C*50*_Propo_* (µg∙mL^−1^)	8.79 (35.3)	7.38 (4.07–12.41)	-
IIV *C*50*_Propo_* (%)	104.0 (20.3)	119.5 (67.8–281.9)	7.5
*Emax_Propo_*	0.88 (22.9)	0.77 (0.54–0.99)	-
*C*50*_Remi_* (ng∙mL^−1^)	4.57 (24.9)	3.63 (1.54–7.61)	-
IIV *C*50*_Remi_* (%)	103.7 (14.7)	97.6 (67.0–169.7)	7.2
*Emax_Remi_*_,*HR*_	0.69 (9.8)	0.65 (0.35–0.81)	-
*γ_Remi_*	1.20 (19.5)	1.37 (0.94–2.65)	-
Cardiovascular system related			
*MAP*_0_ (mmHg)	88.2 (1.3)	88.2 (86.0–90.2)	-
IIV *MAP*_0_ (%)	8.63 (9.1)	8.51 (6.83–10.04)	6.5
*HR*_0_ (bpm)	70.8 (1.9)	70.8 (68.2–73.7)	-
IIV *HR*_0_ (%)	14.4 (11.9)	14.2 (11.4–18.1)	1.0
*k_out_* (min^−1^)	0.123 (14.9)	0.126 (0.091–0.166)	-
IIV *k_out_*_,*MAP*_ (%)	142.6 (12.5)	144.2 (101.6–221.9)	6.7
IIV *k_out_*_,*HR*_ (%)	130.2 (18.9)	128.4 (71.1–262.8)	13.2
*FB_HR_*	0.806 (31.6)	0.842 (0.539–1.526)	-
Stimulus related			
*k_Stim_* (min^−1^)	0.021 (54.8)	0.018 (0.004–0.044)	-
$\theta_{Stim}$	0.284 (16.7)	0.277 (0.186–0.394)	-
Covariance between random effects			
*C*50*_Propo_*, *k_out_*_,*MAP*_ (unitless)	0.76 (31.3)	0.83 (0.42–1.43)	-
Residual variability			
Residual error in *MAP* (mmHg)	5.53 (4.0)	5.51 (5.08–5.94)	3.3
Residual error in *HR* (bpm)	4.30 (5.8)	4.28 (3.80–4.83)	2.5

RSE, relative standard error, is calculated as $\frac{SE}{\bar{\hat{\theta }}}\times 100$, where SE corresponds to the standard error and $\hat{\theta }$ corresponds to the point estimate of the parameter; CIs, confidence intervals, obtained from the five hundred bootstrap analyses; IIV, inter-individual variability, expressed as the coefficient of variation [CV(%)] and calculated as $\sqrt{e^{\omega^{2}}-1}\times 100$, where ω^2^ corresponds to the variance of the random effect.

## Data Availability

Data are unavailable due to privacy restrictions as part of an ongoing study.

## Supplementary Materials

The following supporting information can be downloaded at: https://www.mdpi.com/article/10.3390/pharmaceutics16121615/s1, Table S1: Detailed description of study schemes of propofol and remifentanil; Figure S1: Exploratory graphical analysis of (randomly selected) raw individual profiles; Figure S2: Individual fit profiles of randomly selected patients; Figure S3: Model validation.
