# Supplementary material for: Pharmacodynamic Model of the Hemodynamic Effects of Propofol and Remifentanil and Their Interaction with Noxious Stimulation
Source: Pharmaceutics. 2024 Dec 19;16(12):1615. doi: 10.3390/pharmaceutics16121615 (PMC11677492; doi:10.3390/pharmaceutics16121615)

Table S1. Detail description of the study schemes of propofol and remifentanil.

Group A

ID	Propofol site-effect target (μg·mL^{-1})							
	0 min	5 min	10 min	15 min	20 min	25 min	30 min	35 min
1	1	2	3	4	4	4	4	4
2	1.2	2.4	3.6	4.8	4.8	4.8	4.8	4.8
3	0.8	1.6	2.4	3.2	3.2	3.2	3.2	3.2
4	1.5	3	4.5	4.5	4.5	4.5	4.5	4.5
5	1.7	3.4	5.1	5.1	5.1	5.1	5.1	5.1
6	1.3	2.6	3.9	3.9	3.9	3.9	3.9	3.9
7	2.7	2.7	2.7	2.7	2.7	2.4	2.1	1.8
8	3	3	3	3	3	2.8	2.6	2.4
9	3.3	3.3	3.3	3.3	3.3	2.6	1.9	1.2
10	3.7	3.7	3.7	3.7	3.7	3.2	2.7	2.2
11	4	4	4	4	4	2.8	1.6	1.6
12	4.3	4.3	4.3	4.3	4.3	3.3	2.3	1.3

Group B

ID	Remifentanil site-effect target (ng·mL^{-1})							
	0 min	5 min	10 min	15 min	20 min	25 min	30 min	35 min
1	0.5	1	1.5	2	2	2	2	2
2	0.7	1.4	2.1	2.8	2.8	2.8	2.8	2.8
3	1	2	3	4	4	4	4	4
4	0.8	1.6	2.4	3.2	3.2	3.2	3.2	3.2
5	1.5	1.5	1.5	1.5	1.5	1.0	0.5	0.5
6	2	2	2	2	2	1.7	1.4	1.1
7	2.5	2.5	2.5	2.5	2.5	1.5	0.5	0.5
8	3	3	3	3	3	2.3	1.6	0.9

Group C

ID	Propofol site-effect target (μg·mL^{-1})	Remifentanil site-effect target (ng·mL^{-1})							
	Continuous	0 min	5 min	10 min	15 min	20 min	25 min	30 min	35 min
1	3	1	2	3	4	4	4	4	4
2	2.5	0.8	1.6	2.4	3.2	3.2	3.2	3.2	3.2
3	3.5	0.7	1.4	2.1	2.8	2.8	2.8	2.8	2.8
4	4	0.6	1.2	1.8	2.4	2.4	2.4	2.4	2.4
5	3	0.5	1	1.5	2	2	2	2	2
6	3	1.5	1.5	1.5	1.5	1.5	1.0	0.5	0.5
7	2.7	2	2	2	2	2	1.7	1.4	1.1
8	3.3	2.5	2.5	2.5	2.5	2.5	1.5	0.5	0.5
9	3.7	3	3	3	3	3	1.8	0.6	0.6
10	3.5	3.5	3.5	3.5	3.5	3.5	2.8	2.1	1.4

Group D

ID	Remifentanil site-effect target (ng·mL^{-1})	Propofol site-effect target (μg·mL^{-1})							
	Continuous	0 min	5 min	10 min	15 min	20 min	25 min	30 min	35 min
1	1.5	1.7	3.4	5.1	5.1	5.1	5.1	5.1	5.1
2	2.5	1.3	2.6	3.8	3.8	3.8	3.8	3.8	3.8
3	2.7	0.8	1.6	2.4	3.2	3.2	3.2	3.2	3.2
4	1.8	1	2	3	4	4	4	4	4
5	2	1.5	3	4.5	4.5	4.5	4.5	4.5	4.5
6	1.2	2.7	2.7	2.7	2.7	2.7	2.2	1.7	1.2
7	1.8	3	3	3	3	3	2.8	2.6	2.4
8	2.2	3.3	3.3	3.3	3.3	3.3	2.1	0.9	0.9
9	2.8	3.7	3.7	3.7	3.7	3.7	3.0	2.3	1.6
10	3.2	4	4	4	4	4	3	2	1

Figure S1. Exploratory graphical analysis of (randomly selected) raw individual profiles.
Open circles are raw observations for mean arterial pressure (MAP, in blue) and heart rate (HR, in red). Solid lines correspond to the predicted plasma concentrations (C) of propofol (yellow) and remifentanil (green). Grey area highlights the period of surgery, being discontinuous in one patient.

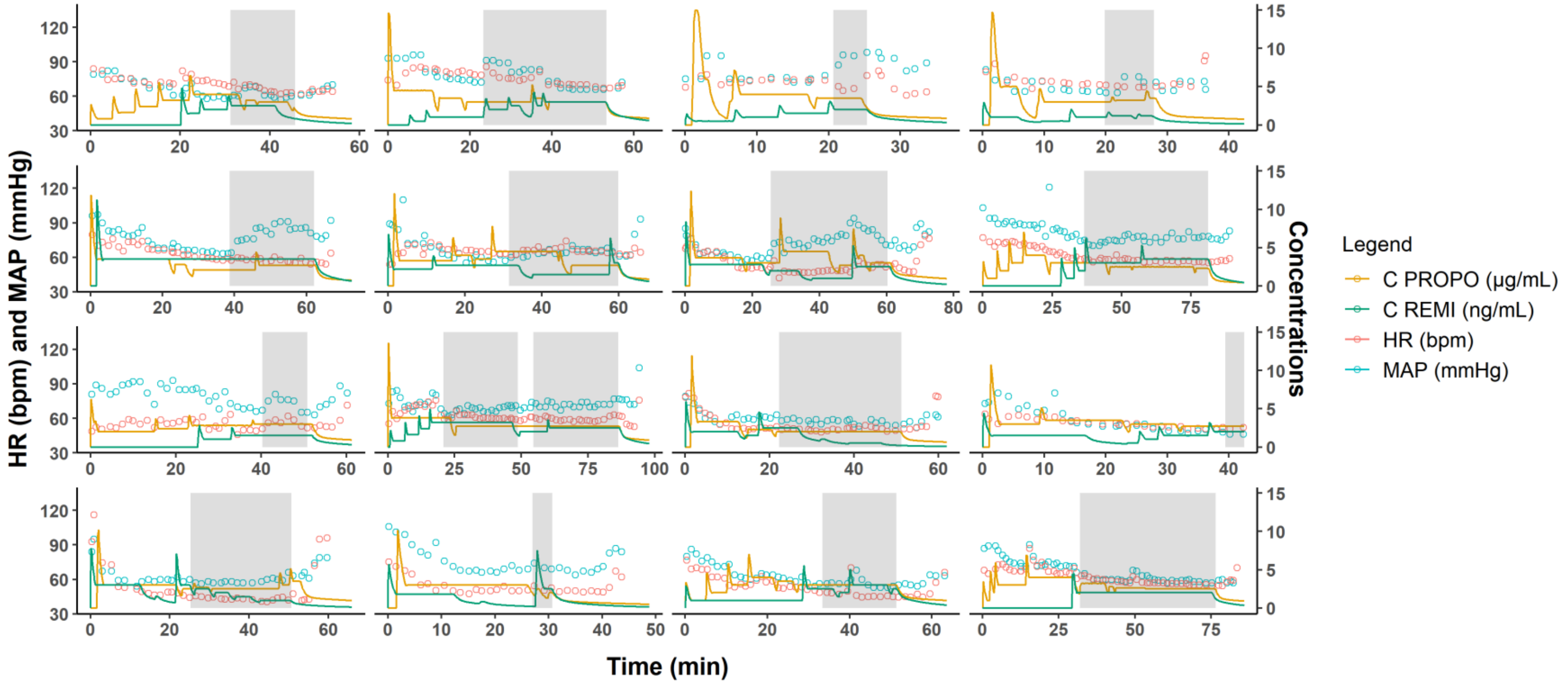

Figure S2. Individual fit profiles of randomly selected patients.

Observations (points) and individual model predictions (solid line), Blue corresponds to MAP response, red to HR response. Grey area highlights the period of surgery, being discontinuous in one patient.

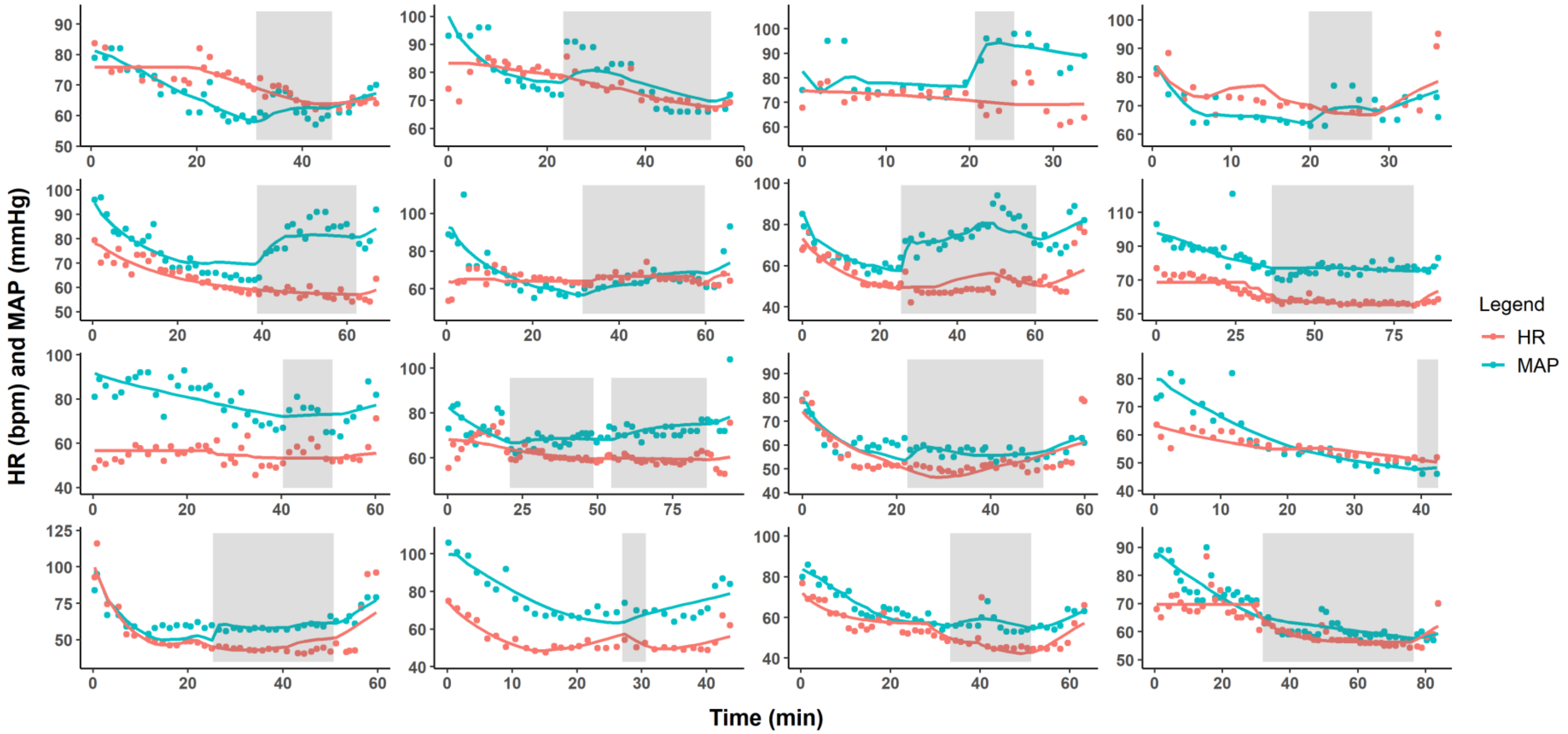

Figure S3. Model validation.

Prediction-corrected visual predictive checks for MAP and HR. The median (solid line), 2.5th and 97.5th percentiles (dashed lines) of raw data are plotted along with the 95% prediction intervals (shaded area) for the median and 2.5th and 97.5th percentiles obtained from 500 simulated datasets. Data before and after the surgical intervention are coloured in brown, while points during the stimulation are coloured in yellow. Validation dataset (test group, corresponding only induction and no-hysteroscopy surgery records) have been superimposed on the pcVPC of the training set (Figure 3C) coloured in green.

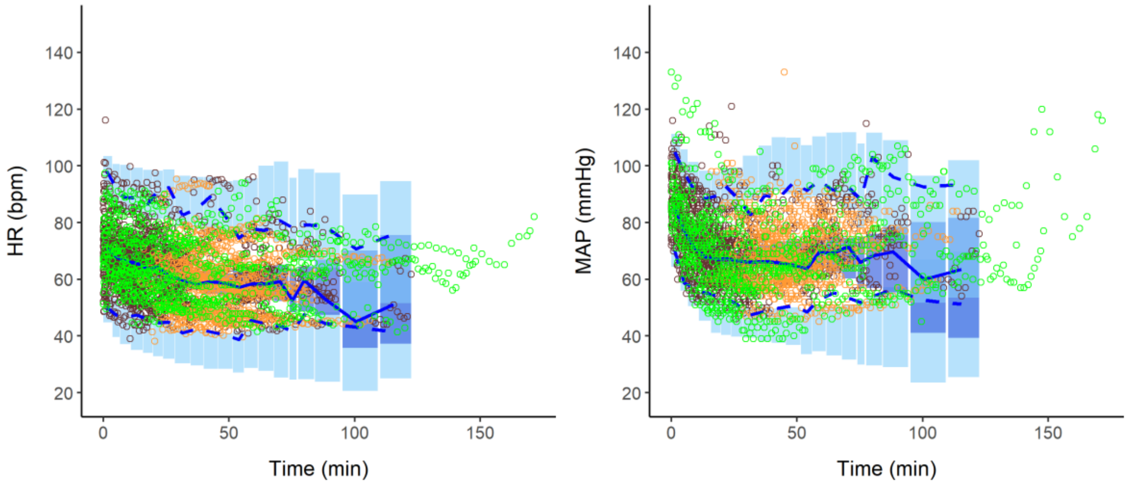

Supplement: Supplementary file 1 [file pharmaceutics-16-01615-s001.zip › pharmaceutics-3305968-supplementary.pdf]
